# Genome-Wide microRNA Binding Site Variation between Extinct Wild Aurochs and Modern Cattle Identifies Candidate microRNA-Regulated Domestication Genes

**DOI:** 10.3389/fgene.2017.00003

**Published:** 2017-01-31

**Authors:** Martin Braud, David A. Magee, Stephen D. E. Park, Tad S. Sonstegard, Sinead M. Waters, David E. MacHugh, Charles Spillane

**Affiliations:** ^1^Genetics and Biotechnology Lab, Plant and AgriBiosciences Research Centre, School of Natural Sciences, National University of Ireland Galway, University RoadGalway, Ireland; ^2^Animal Genomics Laboratory, UCD School of Agriculture and Food Science, University College DublinDublin, Ireland; ^3^IdentiGEN Ltd, Unit 2, Trinity Enterprise CentreDublin, Ireland; ^4^Recombinetics, Inc., St. PaulMN, USA; ^5^Animal and Bioscience Research Department, Animal and Grassland Research and Innovation Centre, TeagascDunsany, Ireland; ^6^UCD Conway Institute of Biomolecular and Biomedical Research, University College DublinDublin, Ireland

**Keywords:** microRNA, polymorphism, evolution, domestication, agriculture, *Bos primigenius*, *Bos taurus*, ancient DNA

## Abstract

The domestication of cattle from the now-extinct wild aurochs (*Bos primigenius*) involved selection for physiological and behavioral traits, with underlying genetic factors that remain largely unknown. Non-coding microRNAs have emerged as key regulators of the spatio-temporal expression of target genes controlling mammalian growth and development, including in livestock species. During the domestication process, selection of mutational changes in miRNAs and/or miRNA binding sites could have provided a mechanism to generate some of the traits that differentiate domesticated cattle from wild aurochs. To investigate this, we analyzed the open reading frame DNA sequence of 19,994 orthologous protein-coding gene pairs from extant *Bos taurus* genomes and a single extinct *B. primigenius* genome. We identified miRNA binding site polymorphisms in the 3′ UTRs of 1,620 of these orthologous genes. These 1,620 genes with altered miRNA binding sites between the *B. taurus* and *B. primigenius* lineages represent candidate domestication genes. Using a novel Score Site ratio metric we have ranked these miRNA-regulated genes according to the extent of divergence between miRNA binding site presence, frequency and copy number between the orthologous genes from *B. taurus* and *B. primigenius.* This provides an unbiased approach to identify cattle genes that have undergone the most changes in miRNA binding (i.e., regulation) between the wild aurochs and modern-day cattle breeds. In addition, we demonstrate that these 1,620 candidate domestication genes are enriched for roles in pigmentation, fertility, neurobiology, metabolism, immunity and production traits (including milk quality and feed efficiency). Our findings suggest that directional selection of miRNA regulatory variants was important in the domestication and subsequent artificial selection that gave rise to modern taurine cattle.

## Introduction

Since their domestication from wild aurochs (*Bos primigenius*) some 10,000 years ago, cattle (*Bos taurus* and *Bos indicus*) have been continuously exposed to both natural and artificial selection (Bradley and Magee, 2006; Magee et al., 2014). These selection processes, coupled with mutation, genetic drift and admixture have produced the multitude of extant humpless taurine and humped zebu cattle breeds (Food and Agriculture Organization, 2015). However, the underlying genetic factors (including genes, regulatory elements and DNA sequence polymorphisms) contributing to phenotypic traits selected during and after cattle domestication remain largely unknown (Elsik et al., 2009; Canavez et al., 2012; Kemper and Goddard, 2012; Womack, 2012; Gutierrez-Gil et al., 2015; Utsunomiya et al., 2015).

Many studies have highlighted the importance of microRNAs (miRNAs)—short non-coding RNAs that post-transcriptionally modulate gene expression—in regulating a wide range of biological processes in vertebrate species, including domestic livestock (McDaneld, 2009; Liu et al., 2010; Fatima and Morris, 2013; Wang et al., 2013). In mammals, miRNA targeting has predominantly been associated with the 3′ UTR region of transcripts derived from open reading frames (ORFs), typically leading to down-regulation through triggering RNA degradation, RNA instability and/or reduced translation (Winter et al., 2009; Krol et al., 2010; Huntzinger and Izaurralde, 2011).

DNA sequence polymorphisms occurring within the targeted miRNA binding site of genes can influence phenotypic traits of economic importance in mammalian livestock (Clop et al., 2006; Sasaki et al., 2013; Hou et al., 2015; Rong et al., 2015; An et al., 2016). Hence, we hypothesized that DNA sequence polymorphisms affecting miRNA-mediated gene regulation are likely associated with pre- and post-domestication differences in cattle, particularly for traits under natural and artificial selection within the ∼10,000 year time frame of cattle domestication (Larson and Fuller, 2014; Larson et al., 2014; MacHugh et al., 2016).

Using sequence data from the recently published *B. primigenius* genome (Park et al., 2015) and sequenced genomes of modern-day *B. taurus* (Zimin et al., 2009), we developed a ranking metric [Score Site ratio (SSr)] to identify the miRNA-targeted bovine genes that display the greatest sequence difference in their miRNA binding sites between domesticated taurine cattle breeds and wild aurochs. Using this approach we have identified genes displaying high levels of miRNA binding site DNA sequence differences (polymorphisms) between orthologous gene pairs where one gene is from the wild aurochs and the other is from domesticated taurine cattle. Genes displaying post-domestication variation in miRNA binding sites are shown to be involved in mammalian growth and development (e.g., ephrin signaling and androgen signaling) and cellular function and metabolism (e.g., cholesterol biosynthesis). We consider that some of the miRNA binding site polymorphisms identified here, which are either absent or specific to the aurochs genome, modulate expression of candidate domestication genes associated with traits that have been selected during or subsequent to domestication.

## Materials and Methods

### Aurochs Genome Sequence Data

All *B. primigenius* genome sequence data was retrieved from the Sequence Read Archive database (accession number SRX1266623). The laboratory and bioinformatics methods used for DNA extraction, purification and sequence analysis of the CPC98 aurochs specimen have been described by us previously (Park et al., 2015). The genome sequence data is from an archeologically verified *B. primigenius* specimen retrieved in 1998 from Carsington Pasture Cave in Derbyshire, England, and radiocarbon dated to 6,738 ± 68 calibrated (cal.) years before present (yBP). The authenticity of the CPC98 genome sequence was verified using a number of different approaches that are detailed by Park et al. (2015). The high-quality single nucleotide polymorphisms (SNPs) that were used for the analyses described here were identified using a stringent computational workflow fully described in Park et al. (2015).

### Identification of *B. primigenius* 3′ UTR Sequences

To identify miRNA binding sites in target genes, 3′ UTR sequences and associated bovine genome coordinates were obtained from Ensembl using the Biomart retrieval tool (Kinsella et al., 2011) and the *B. taurus* UMD3.1 reference genome assembly (Zimin et al., 2009). 3′ UTR sequence information from *B. taurus* UMD3.1 annotation has been used to predict and retrieve the 3′ UTR sequences from the *B. primigenius* genome consensus sequence, using the coordinates associated with each *B. taurus* 3′ UTR. All *B. taurus* and *B. primigenius* 3′ UTR pairs were then used to identify sequence polymorphisms within putative miRNA binding sites for *B. taurus* and *B. primigenius*. In addition, the SNP data from the sequencing of 49 *B. taurus* cattle samples from the Park et al. (2015) aurochs genome study have been used to confirm the fixation of the SNPs identified between *B. taurus* and *B. primigenius* across different breeds (Angus, Holstein, Jersey, Limousin, Romagnola, Fleckvieh, N’Dama).

### Identification of miRNA Complementary Binding Sites in 3′ UTR of Target Genes Using TargetScan

To identify miRNA target binding sites in *B. taurus* and *B. primigenius* 3′ UTR sequences, the TargetScan 6.1 software package was used (Lewis et al., 2005). Targetscan has been previously shown to be an accurate predictor of miRNA binding sites (Baek et al., 2008; Ulitsky et al., 2010). The prediction score for each miRNA binding site incorporates the evolutionary conservation of a predicted miRNA binding site among different species (Friedman et al., 2009). It also takes into account the presence of many structural motifs in mRNA sequences related to miRNA binding to infer a particular score for each site.

The TargetScan 6.1 package consists of two main Perl scripts: targetscan_60.pl and targetscan_60_context_scores.pl. The first script, targetscan_60.pl, identifies only the seed region recognition motif composed of six nucleotides (positions 2–7 in the mature miRNA) and one additional flanking nucleotide (position 1 or 8 of the mature miRNA) to identify four types of seed region [six nucleotides, seven nucleotides (6 + 1 adenosine), seven nucleotides, and eight nucleotides (7 + 1 adenosine)]. The script targetscan_60.pl takes as its input the sequence alignments of the 3′ UTRs and the list of miRNA seed regions for *B. taurus* and *B. primigenius* miRNAs. For each alignment the script returns an output containing the miRNA seed regions that have been identified in the 3′ UTR and their corresponding positions. The output of this script was parsed using a custom Python script that reads through the output files and extracts the information contained in each line of the file. This includes the binding site nucleotide location in the 3′ UTR and the group of species that have a site at the same position in the 3′ UTR multiple alignments. *B. taurus* and *B. primigenius* seed regions that were identified, yet contained ambiguous nucleotides in the *B. primigenius* 3′ UTR were removed from the analysis. Any seed sites overlapping with a previously identified seed site for the same miRNA were also removed.

The second Targetscan Perl script, targetscan_60_context_scores.pl, generates a score for each site taking into account the position of the seed region in the 3′ UTR, its nucleotide composition, the pairing of the miRNA in its 13–16 position with the UTR and the composition of the flanking region of the binding site. The Targetscan context score corresponds to a prediction, based on experimental miRNA repression expressed as log-ratios (Grimson et al., 2007; Garcia et al., 2011). For this script, the results files from the first Targetscan script (cataloged as either a gain, a loss, an increase or a decrease of miRNA target sites), the alignments and the list of mature miRNA sequences specific to either *B. taurus* or *B. primigenius* were used as inputs. To parse the results, a custom Python script was used to generate two results files: a file containing genes with miRNA binding sites polymorphic between aurochs and domestic taurine cattle and a second file with shared miRNA binding sites across the two taxa. All results in which ambiguous nucleotides were present in the UTR target region (complementary to the 3′ end region of the mature miRNA) were removed. For miRNAs with multiple binding sites, the score for each gene/miRNA pair was generated by summing the individual context scores (calculated by TargetScan for each site).

To rank the targeted genes, we calculated an additional score, termed the *SSr*), which facilitated analysis of the extent to which miRNA binding sites were polymorphic between *B. taurus* and *B. primigenius*, relative to those that were common to both *B. taurus* and *B. primigenius*. The *SSr* was calculated by summing all context scores for each predicted miRNA binding site across each 3′ UTR and multiplying this sum by the ratio of the number of polymorphic sites over the number of shared sites between *B. taurus* and *B. primigenius*. The *SSr* statistic is calculated using the following equation:

$$\begin{array}{c} SSr=\sum_{i\;=\;1}^{n}score_{i}\times \frac{psn}{csn} \end{array}$$

where *n* is the number of miRNA binding sites identified for a particular gene UTR; score*_i_* is the score for each predicted site; *psn* represents the number of miRNA binding sites polymorphic between *B. taurus* and *B. primigenius* for a particular gene; and *csn* represents the number of miRNA binding sites common to *B. taurus* and *B. primigenius* for a particular gene.

The *SSr* score was used to rank genes according to the extent of their miRNA site polymorphisms differentiating *B. primigenius* and *B. taurus*. This provided a basis for ranking of the genes according to the extent of dysregulation of their miRNA binding sites between aurochs and modern cattle. In addition, the *SSr* statistic provides a combined weighting for each gene, which combines both the number of different miRNA binding sites (ratio of number of sites) and their possible impact (sum of TargetScan scores) between *B. taurus* and *B. primigenius* and facilitates normalization of the number of discovered sites, which can depend of the size of the 3′ UTR.

### Analyses of Biological Pathways Involved in Differential miRNA Targeting of *B. taurus* Genes Relative to *B. primigenius* Genes

To identify the biological pathways that are most affected by miRNA binding site variation between the 3′ UTRs of *B. taurus* and *B. primigenius* genes, Ingenuity Systems Pathway Analysis (IPA – www.ingenuity.com) was used. *B. taurus* genes with variant miRNA binding sites (when compared with *B. primigenius*) were used as the input to IPA. The IPA core analysis evaluates enrichment of the selected genes in different pathways, returning a *P*-value for each pathway identified, which was then used to rank enriched pathways and identify the most statistically significant (*P*-value < 0.05). A pathway score was then assigned to each significant pathway corresponding to the sum of the *SSr* score for each of the genes associated with a particular pathway. Significant pathways were then assigned a summed *SSr* score to produce a ranked list of the pathways containing genes with significant miRNA binding site differences between *B. taurus* and *B. primigenius*.

### Analysis of Transition/Transversion Ratios

To determine the transition/transversion ratio (ti/tv) in the 3′ UTR miRNA binding site SNPs, the fixed SNPs identified in the miRNA binding sites were sorted into two mutational classes based on the variants observed: a transition class (A↔G, C↔T) and a transversion class (A↔C, A↔T, C↔G, G↔T). Using a custom python script, the number of variants were summed for each group and the ti/tv ratio was calculated.

## Results

### DNA Sequence Differences between the 3′ UTRs of *B. taurus* and *B. primigenius* Genes

Of the 19,994 annotated protein-coding genes in the taurine cattle genome (assembly UMD3.1), 11,761 have an annotated 3′ UTR (59%). To identify all SNPs differentiating the 3′ UTRs of *B. taurus* and *B. primigenius* genes, we compared these 11,761 3′ UTR sequences from *B. taurus* with the corresponding mapped regions in *B. primigenius*. We determined that 3,066 (26%) of these genes contained one or more SNPs, and identified a total of 6,355 SNPs that were different between *B. taurus* and *B. primigenius* in the 3,066 3′ UTRs. Analysis of the numbers of SNPs in the 3′ UTR of each gene yielded a mean of 1.92 SNPs per 3′ UTR, with a median value of 1.00 SNPs per 3′ UTR and a maximum of 27 *B. taurus*/*B. primigenius* SNPs for the methionine adenosyltransferase I, alpha gene (*MATA1*).

### DNA Sequence Polymorphisms Differentiating *B. taurus* and *B. primigenius* 3′ UTRs Generate Altered miRNA Binding Sites

Alteration of miRNA binding sites in 3′ UTRs of target genes can disrupt the regulatory effects of existing miRNAs, and/or can generate new miRNA binding sites. To investigate this further, we screened for SNPs that differentiate the 3′ UTRs of *B. taurus* and *B. primigenius* genes. From the 3,066 3′ UTRs exhibiting SNPs differentiating the two taxa, we identified a total of 2,634 SNPs located in miRNA binding sites within 1,620 genes. Notably, DNA sequence polymorphisms in the 3′ UTR of the protein kinase, AMP-activated, gamma 3 non-catalytic subunit gene (*PRKAG3*) produced the highest number of miRNA binding site changes across all of these 1,620 genes. For the *PRKAG3* 3′ UTR, our analysis demonstrated that 23 different miRNAs have a modification of their binding potential (gain or loss). These 23 miRNAs were associated with 15 distinct miRNA-binding sites that differ between the *B. taurus* and *B. primigenius PRKAG3* genes.

For our analysis of polymorphisms in 3′ UTRs, individual miRNA binding sites were classified as a “gain” when SNPs between *B. taurus* and *B. primigenius* generate a new miRNA binding sites in *B. taurus* 3′ UTRs, and as a “loss” when they lead to loss of an existing miRNA binding site in the *B. taurus* genome. In some cases, a polymorphic miRNA binding site can be considered simultaneously as a “gain” and a “loss” when one or more SNPs generate new binding sites for one particular miRNA and eliminate a binding site for another miRNA. An additional scenario we considered is when a miRNA gains a new binding site in *B. taurus* but also has another common binding site between *B. taurus* and *B. primigenius*. We categorize such miRNA binding site copy number “amplification” as an “increase,” while a reduction of binding site copy number it is categorized as a “decrease.”

**FIGURE 1:**
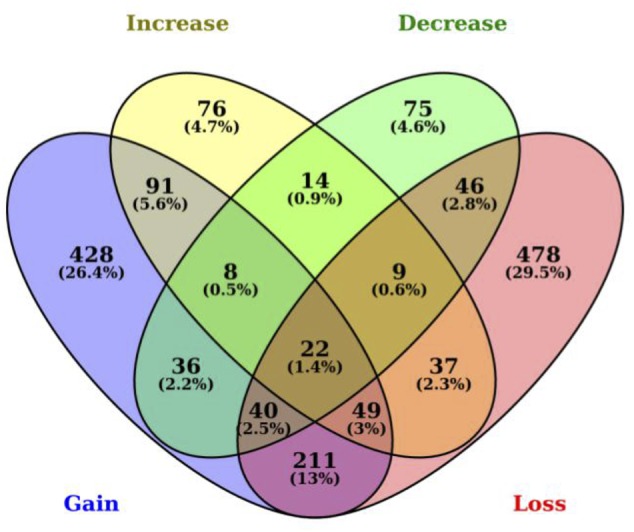
**Polymorphisms between *Bos taurus* and *Bos primigenius* in 3′ UTRs generate or disrupt miRNA binding sites.** Venn diagram representing the number of genes with 3′ UTR polymorphisms between *B. taurus* and *B. primigenius*. MiRNA binding sites are represented as gained (blue), lost (yellow), increased (green), or decreased (red). The intersection represents SNPs in 3′ UTRs that generate a binding site for one miRNA while disrupting another miRNA binding site in the same gene (within the same miRNA-binding site region).

Among the 1,620 genes with SNPs in 3′ UTR miRNA binding sites, we determined that 892 genes have lost their miRNA binding sites including up to 13 losses for the membrane-spanning four-domains, subfamily A, member seven gene (*MS4A7*) in the *B. taurus* lineage. We also identified 885 genes that have gained a miRNA binding site in *B. taurus*, which is not present in *B. primigenius* including a gain of up to 11 new miRNA binding sites for the 3-hydroxy-3-methylglutaryl-CoA synthase 2 gene (*HMGCS2*). There is an overlap of 322 genes that have both a gain and loss of miRNA binding sites in *B. taurus*. We also determined that 306 genes in *B. taurus* have a number of miRNA-binding sites that have increased in copy number for at least one miRNA (up to seven miRNA binding sites for the ubiquitin specific peptidase 33 gene, *USP33*), while 250 genes have a decrease in miRNA binding site copy number up to 11 miRNA binding sites for the *MAT1A* gene in comparison to *B. primigenius* (**Figure 1**; Supplementary Table S1).

### Using SSr Scores to Rank the 3′ UTRs that have undergone the Most Extensive miRNA Binding Site Modifications between *B. primigenius* and *B. taurus*

We hypothesized that selection processes (both natural and artificial) during and after the domestication of cattle may have led to new allelic variants at miRNA binding sites of genes that are associated with anatomical, physiological and behavioral trait variation in cattle. To identify miRNA-targeted genes (and associated cellular pathways) that displayed the most miRNA targeting divergence between *B. taurus* and *B. primigenius*, we used the *SSr* score metric to rank all of the genes. Several of the top 10 *SSr*-ranked genes were found to be involved in key metabolic and physiological processes. These included: (1) the guanylate cyclase activator 2B gene (*GUCA2B*), in which the binding sites for five miRNAs (bta-miR-541, bta-miR-2433, bta-miR-1777a, bta-miR-2454, and bta-miR-2327) were absent in *B. taurus* but present in *B. primigenius*, with only one common binding site between both; (2) the *HMGCS2* gene, in which the binding sites for 11 miRNAs were present in *B. taurus* but absent in *B. primigenius* and also the number of binding sites increased for two miRNAs in *B. taurus*; and (3) the *MS4A7* gene, in which the binding sites for 13 miRNAs were absent in *B. taurus* and present in *B. primigenius*, which represented the most significant loss of potential miRNA binding sites in our entire data set.

We also identified three genes important for metabolism: the glutathione peroxidase 5 gene (*GPX5*), the casein kappa gene (*CSN3*) and the *MAT1A* gene, which displayed DNA sequence differences between the *B. taurus* and *B. primigenius* lineages in their respective 3′ UTR miRNA binding sites (**Table 1**).

**Table 1:** Genes with polymorphic miRNA binding sites in candidate miRNA-regulated cattle domestication genes.

Gene	Rank	SSr	miRNA targeting polymorphic sites	Type and no. of events
*GUCA2B*	1	-5.200	bta-miR-541:bta-miR-2433:bta-miR-1777a:bta-miR-2454:bta-miR-2327	Loss:5
*MS4A7*	6	-1.156	bta-miR-17-5p:bta-miR-2284p:bta-miR-221:bta-miR-222:bta-miR-93:bta-miR-20b:bta-miR-433:bta-miR-20a:bta-miR-302d:bta-miR-106:bta-miR-302b:bta-miR-302c:bta-miR-106b	Loss:13
*HMGCS2*	9	-0.905	bta-miR-301b:bta-miR-301a:bta-miR-2375:bta-miR-454:bta-miR-152:bta-miR-148b:bta-miR-148a:bta-miR-130a:bta-miR-130b:bta-miR-2317:bta-miR-2329-3p:bta-miR-199a-5p:bta-miR-199b	Increase:2 Gain:11
*PRKAG3*	16	-0.659	bta-miR-2302:bta-miR-345-5p:bta-miR-2311:bta-miR-2309:bta-miR-2329-3p:bta-miR-542-5p:bta-miR-2917:bta-miR-141:bta-miR-2330:bta-miR-200a:bta-miR-1343:bta-miR-2397:bta-miR-429:bta-miR-2285b:bta-miR-200c:bta-miR-200b:bta-miR-491:bta-miR-2888:bta-miR-873:bta-miR-2383:bta-miR-764:bta-miR-369-3p:bta-miR-2442	Increase:6 Decrease:1 Gain:6 Loss:10
*GPX5*	30	-0.298	bta-miR-677:bta-miR-449d:bta-miR-26c:bta-miR-33a:bta-miR-2294:bta-miR-33b:bta-miR-18b:bta-miR-18a:bta-miR-217:bta-miR-2461-5p:bta-miR-2319b	Increase:1 Gain:8 Loss:2
*MAT1A*	32	-0.293	bta-miR-2459:bta-miR-495:bta-miR-423-3p:bta-miR-2388:bta-miR-2406:bta-miR-2427:bta-miR-2450b:bta-miR-194:bta-miR-2888:bta-miR-2327:bta-miR-491:bta-miR-152:bta-miR-148b:bta-miR-1777a:bta-miR-148a:bta-miR-2454:bta-miR-2433:bta-miR-2416:bta-miR-1434	Increase:1 Decrease:11 Gain:2 Loss:5
*CSN3*	55	-0.193	bta-miR-2312:bta-miR-2301:bta-miR-1603:bta-miR-2337	Gain:1 Loss:3

### Identification of Networks, Pathways, and Biological Functions Enriched in Genes with miRNA Target Sites Polymorphic between *B. taurus* and *B. primigenius*

We used Ingenuity Systems Pathway Analysis (IPA) to gain further insight into the biological functions, canonical pathways and biological networks enriched for genes with miRNA binding sites polymorphic between *B. taurus* and *B. primigenius*. For this, we uploaded the 1,620 genes identified by *SSr* analysis and ranked the IPA-identified functions, canonical pathways and networks based on enrichment *P*-values generated by IPA. Using this approach, we identified 25 networks, 74 pathways, and 500 functions that are enriched for genes with miRNA binding sites polymorphic between *B. taurus* and *B. primigenius*.

**FIGURE 2:**
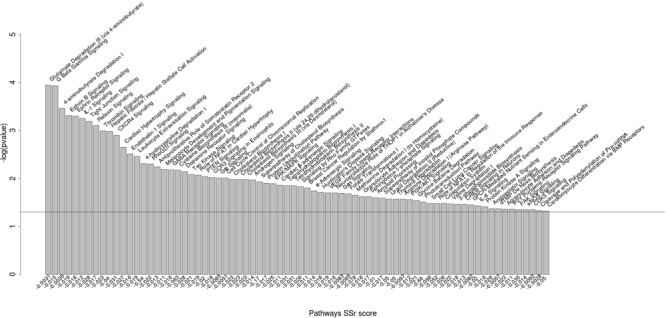
**Biological pathways significantly enriched for miRNA polymorphic target genes.** Biological pathways significantly enriched for genes having SNPs in miRNA binding sites between *B. primigenius* and *B. taurus* [*P*-value < 0.05; -log_10_(*P*-value) > 1.3]. The pathways significantly enriched are represented by –log_10_(*P*-value) in the ordinate. The ratio of genes corresponds to the number of candidate genes over the number of background genes in one pathway (ranked by *P*-value and *SSr* score).

The two top-ranked networks we identified were metabolism and neurological diseases. Among the significantly enriched canonical pathways differentially regulated by the miRNAs were pathways involved in immunobiology (for example, the IL-1, CXCL4, and NFAT pathways), physiology and development (for example, the ephrin signaling pathway and the PTEN and PI3K/AKT signaling pathways), and reproductive physiology (for example, the androgen signaling pathway) (**Figure 2**).

### The Polymorphic miRNA Binding Sites in Aurochs 3′ UTRs are not Enriched for Transitions due to Post-mortem Deamination

The analysis of ancient DNA sequence data can be complicated where post-mortem deamination leading to C→T or G→A transition substitutions occurs (Hofreiter et al., 2001; Briggs et al., 2007). To investigate whether the 2,634 SNPs (that were polymorphic in the miRNA binding sites in aurochs 3′ UTRs) could be enriched for transitions due to post-mortem deamination, the ti/tv ratio was calculated for the set of 2,634 SNPs located in miRNA binding sites and was determined to be 1.93:1.00. Previously, for the entire CPC98 aurochs genome, we examined 2,009,261 biallelic SNPs (73.3% of which were homozygous) and determined a transition to transversion (ti/tv) ratio of 2.19:1.00 (Park et al., 2015), which is very similar to ti/tv ratios obtained for female and male Holstein genome sequences: 2.18:1.00 and 2.16:1.00, respectively (Koks et al., 2013, 2014). The lower ti/tv ratio detected for the 3′ UTR miRNA SNPs is consistent with published ti/tv ratios calculated for mammalian 3′ UTRs (Chen et al., 2015), and suggests that post-mortem deamination is not a confounding factor for SNPs within miRNA binding sites located in aurochs 3′ UTRs.

## Discussion

Analyses of whole genome sequence data from mammalian livestock (and domesticated companion animals) and their wild ancestors have begun to reveal microevolutionary processes, key genetic variants and biological pathways underlying animal domestication (Orlando et al., 2013; Schubert et al., 2014; Der Sarkissian et al., 2015; Librado et al., 2015; Park et al., 2015; Skoglund et al., 2015; Frantz et al., 2016; MacHugh et al., 2016). MicroRNAs play key roles in regulating a wide range of biological processes at the post-transcriptional level. Indeed, DNA sequence variation in miRNA-binding sites of mammalian genes can have a profound influence on phenotypic variation.

In this study, we hypothesized that genome-wide comparison of miRNA-binding sites in the 3′ UTR of genes of domestic *B. taurus* with miRNA binding sites in the 3′ UTR of orthologs in the wild ancestor, *B. primigenius*, would identify which genes have undergone the most change in relation to miRNA-regulation between wild and domesticated cattle. We consider that genes which have undergone the most divergence in miRNA binding sites between wild and domestic cattle represent candidate miRNA-regulated domestication loci, which may be associated with phenotypic traits subject to selection during and after the domestication process.

Our analysis of *B. taurus* and *B. primigenius* genome sequences has identified 2,634 SNPs in the 3′ UTR miRNA binding sites of 1,620 bovine genes that differentiate domestic cattle and the wild aurochs. These SNPs were demonstrated to have a low ti/tv ratio, indicating that post-mortem cytosine deamination is not a significant confounding factor in establishing the veracity of these sequence polymorphisms. To identify the 3′ UTRs that have undergone the greatest extent of miRNA binding site divergence between *B. taurus* and *B. primigenius* we developed a novel *SSr* metric. Using the *SSr* ranking approach we have identified candidate miRNA-regulated domestication genes and pathways with roles in immunology, metabolism, physiology, development and fertility.

### Neurological Development Functions

Modifications to neurological development likely led to tameness and facilitated human handling of cattle, for instance through increased docility (Jensen, 2014; Jensen and Wright, 2014). In our previous study of the *B. primigenius* genome, we demonstrated that the bovine phytanoyl-CoA 2-hydroxylase interacting protein gene (*PHYHIP*), which is involved in neurodevelopment, is targeted differentially by a miRNA gene variant (i.e., not a miRNA binding site variant in the target gene) that is polymorphic between aurochs and domestic cattle (Park et al., 2015). In this study, we further demonstrate that Eph/ephrin intracellular signaling pathways, involved in brain development (Lai and Ip, 2009; Hruska and Dalva, 2012; Cramer and Miko, 2016), are enriched in genes with miRNA target sites that are highly polymorphic between aurochs and cattle (**Figure 2**). Given the selection during domestication for behavioral traits (such as docility), we consider that variation in the mRNA-based regulation (i.e., both miRNA genes and miRNA binding sites in 3′ UTRs) of these neurological genes could have been subject to artificial selection.

### Metabolism Functions

Metabolism is an important biological process that likely was modified during and after domestication, as cattle adapted to new diets and feeding regimens associated with increasingly sophisticated animal husbandry practices (Noe-Nygaard et al., 2005). In this context, we have identified three genes and two pathways, which are related to metabolism and have undergone extensive miRNA-based regulation modification in *B. taurus* relative to *B. primigenius*. For instance, the 3-hydroxy-3-methylglutaryl-CoA synthase 2 gene (*HMGCS2*) is targeted by miRNAs in *B. taurus* but not in *B. primigenius*, and is ranked in the top 10 genes by *SSr* score (**Table 1**). The *HMGCS2* protein product is a mitochondrial enzyme involved in ketone metabolism, which varies depending on the availability of dietary carbohydrates (Kostiuk et al., 2010). We also identified miRNA binding site differences between *B. taurus* and *B. primigenius* in the *MAT1A* gene, which is expressed exclusively in mature mammalian liver cells where it is involved in regulation of hepatic function (Mato et al., 2002). The *MAT1A* gene product also plays a role in *S*-adenosylmethionine formation, which has been linked in knockdown mice to hepatic regeneration (Chen et al., 2004). Polymorphisms in *MAT1A* have also been associated with levels of homocysteine in dietary fatty acids (Huang et al., 2012).

Our *SSr*-ranking approach also demonstrated that the *PRKAG3* gene displays extensive miRNA binding site polymorphisms between *B. taurus* and *B. primigenius*. Previous work has shown that *PRKAG3* sequence variants have been associated with differences in porcine skeletal muscle glycogen levels (Milan et al., 2000) and also meat production and quality traits in both domestic pigs and cattle (Reardon et al., 2010; Ryan et al., 2012; Uimari and Sironen, 2014; Zhang et al., 2015; Bongiorni et al., 2016). Finally, the insulin receptor signaling and valine degradation pathways, both of which are associated with metabolism, are also highly enriched for genes with miRNA binding site polymorphisms between *B. taurus* and *B. primigenius*. We consider that differential miRNA-regulatory evolution of metabolic genes and pathways between *B. taurus* and *B. primigenius* may underlie adaptations to changing diets under captivity and animal husbandry.

### Immunological Functions

Under most models of animal domestication, increased confinement and interaction with humans results in increased exposure of early domesticates to novel pathogens, potentially leading to increased selection pressure on genes regulating immune functions (Cleaveland et al., 2001; Harper and Armelagos, 2013; Reperant et al., 2013). In this context, we discovered that the *MS4A7* gene is the gene that has lost the most miRNA binding sites in domestic taurine cattle compared to aurochs. *MS4A7* encodes a member of the membrane-spanning 4A (MS4A) protein family (Eon Kuek et al., 2016), which is expressed in B lymphocytes (Pant et al., 2006; Zuccolo et al., 2013). We have also identified an enrichment of genes with miRNA binding site variants belonging to the nuclear factor of activated T-cells (NFATs) pathway, which is involved in the establishment of innate immune responses to infection (Fric et al., 2012; Zanoni and Granucci, 2012). It is possible that the changes in miRNA regulation of these immune genes between wild and domesticated cattle could be a consequence of domestication and subsequent natural and artificial selection.

### Reproduction, Fertility, and Lactation Functions

Reproduction, fertility, and related biological processes (such as lactation) have been profoundly affected by domestication and human-mediated artificial selection of livestock (Larson and Fuller, 2014; Larson et al., 2014; Zeder, 2015). In this context, we identified two genes and four biological pathways that are ranked highly by *SSr* score (**Table 1**). For instance, *GPX5* is ranked 28th and is targeted by eight additional miRNAs in domestic taurine cattle compared to aurochs. The GPX5 protein plays an important role in the maintenance of spermatozoa DNA integrity (Chabory et al., 2010). *GUCA2B* which encodes guanylate cyclase activator 2B (uroguanylin), is our highest ranked gene with five less miRNA binding sites in domestic cattle compared to aurochs. GUCA2B is an activator of guanylate cyclase (encoded by *GUCY2C*), a membrane protein that modulates cyclic guanosine monophosphate (cGMP) levels, thereby regulating water homeostasis in the intestine and kidneys (Kita et al., 1994; Sindic and Schlatter, 2006). Guanylate cyclase also has a well-characterized role in vasodilatation and blood pressure regulation (Buys and Sips, 2014). Furthermore, a microarray analysis identified variation of *GUCA2B* expression levels in mice and shows that this variation has an effect on pregnancy and fertility (McConaha et al., 2011).

The androgen signaling pathways, which were enriched in genes containing miRNA-binding site polymorphisms between domestic taurine cattle and aurochs, also have a significant impact on both male and female fertility (Smith and Walker, 2014; Walters, 2015). In addition to these two genes, we determined that five pathways related to fertility and reproduction are enriched in genes displaying miRNA binding site variations between *B. taurus and B. primigenius.* The PTEN and PI3K/AKT signaling pathways display significant enrichments that could be linked to fertility. Conjugated linoleic acid has been shown to increase the expression of *PTEN* and *PI3K*/*AKT* regulating granulosa cell proliferation and steroidogenesis in buffaloes, which could affect ovulation and therefore fertility (Sharma and Singh, 2012). Another significantly enriched pathway, the IL-8 pathway is activated during theca cell differentiation and in pre-ovulatory follicle differentiation (Walsh et al., 2012). Finally, one of the highest ranked genes showing loss of miRNA targets in domestic taurine cattle (rank 52/1,606), *CSN3*, encodes casein kappa, a protein associated with the composition and nutritional properties of milk (Caroli et al., 2009; Holt et al., 2013; Pereira, 2014).

### Conclusion

It is important to note that the British *B. primigenius* genome used for this study, although having some admixture with modern-day cattle, is not the main contributor to European cattle breeds (as the major progenitor is known to be *B. primigenius* from the Fertile Crescent domestication center). As we only had one genome of aurochs available we compared this genome to multiple cattle genomes. As more aurochs genome sequence data becomes available some of the miRNA variants identified here as specific to the aurochs lineages may be found to be present in other aurochs genomes (e.g., in aurochs from the Fertile Crescent). Nonetheless, our study provides the first reference point for ongoing and future investigations of the contribution of miRNA variants to cattle domestication.

Overall, we identify a range of candidate miRNA-regulated domestication genes that may underlie some of the phenotypic traits that distinguish domesticated taurine cattle (*B. taurus*) from wild aurochs (*B. primigenius*). These genes have significant miRNA binding site polymorphism divergence between both taxa, which we propose could influence neurobiological, metabolic, immunobiological and reproductive phenotypes associated with the transition from wild aurochs to domesticated taurine cattle.

## Author Contributions

Conceived and designed the project: CS, MB. Managed and oversaw research project: CS. Provided the Aurochs genome sequence and SNP data: SP, DAM, DEM, TS. Analyzed SNPs in miRNA binding site: MB. Wrote the paper: MB, CS. Discussed and edited the paper: CS, MB, DAM, SP, TS, SW, DEM.

## Conflict of Interest Statement

The authors declare that the research was conducted in the absence of any commercial or financial relationships that could be construed as a potential conflict of interest.

The reviewer DN declared a shared affiliation, though no other collaboration, with one of the authors TS to the handling Editor, who ensured that the process nevertheless met the standards of a fair and objective review.
