# Supplementary material for: Genome-Wide microRNA Binding Site Variation between Extinct Wild Aurochs and Modern Cattle Identifies Candidate microRNA-Regulated Domestication Genes
Source: Front Genet. 2017 Jan 31;8:3. doi: 10.3389/fgene.2017.00003 (PMC5281612; doi:10.3389/fgene.2017.00003)
Supplement: Supplementary file 1 [file Data_Sheet_1.docx]

Supplementary Material

Genome-wide microRNA binding site variation between extinct wild aurochs and modern cattle identifies candidate microRNA-regulated domestication genes

Martin Braud^1^, David A. Magee^2^, Stephen D. E. Park^3^, Tad S. Sonstegard^4^, Sinead M. Waters^5^, David E. MacHugh^2, 6^, Charles Spillane^1^*

^1^ Plant and AgriBiosciences Research Centre (PABC), School of Natural Sciences, National University of Ireland Galway, University Road, Galway H91 REW4, Ireland.

^2^ Animal Genomics Laboratory, UCD School of Agriculture and Food Science, University College Dublin, Belfield, Dublin D04 V1W8, Ireland.

^3^ IdentiGEN Ltd, Unit 2, Trinity Enterprise Centre, Pearse Street, Dublin D02 X020, Ireland.

^4^ UCD Conway Institute of Biomolecular and Biomedical Research, University College Dublin, Dublin D04 V1W8, Ireland.

^5^Animal and Bioscience Research Department, Animal and Grassland Research and Innovation Centre, Teagasc, Dunsany, Co. Meath, Ireland.

^6^ UCD Conway Institute of Biomolecular and Biomedical Research, University College Dublin, Dublin D04 V1W8, Ireland.

*** Correspondence:** Charles Spillane, Plant and AgriBiosciences Research Centre (PABC), School of Natural Sciences, National University of Ireland Galway, University Road, Galway H91 REW4, Ireland.
charles.spillane@nuigalway.ie

# Supplementary Figures and Tables

## Supplementary Tables

**Supplementary Table 1**: **Ranked genes with miRNA polymorphic binding sites.**

List of all genes having polymorphic binding sites between *B. primigenius* and *B. taurus*. Rankings are based on the SSR score.
